# Thoracolumbar fascia injury in osteoporotic vertebral fracture: the important concomitant damage

**DOI:** 10.1186/s12891-023-06280-6

**Published:** 2023-03-06

**Authors:** Zicheng Deng, Tao Feng, Xiexing Wu, Haifeng Xie, Dawei Song, Jinning Wang, Huilin Yang, Junjie Niu

**Affiliations:** The authors are from the Department of Orthopaedic Surgery, the First Affiliated Hospital of Soochow University, NO. 899, Pinghai Road, Suzhou, 215006 Jiangsu China

**Keywords:** Osteoporotic vertebral fracture, Thoracolumbar fascia injury, Residual back pain, Percutaneous kyphoplasty, Trauma

## Abstract

**Background:**

Thoracolumbar fascia injury (FI) is rarely discussed in osteoporotic vertebral fracture (OVF) patients in previous literature and it is usually neglected and treated as an unmeaning phenomenon. We aimed to evaluate the characteristics of the thoracolumbar fascia injury and further discuss its clinical significance in the treatment of kyphoplasty for osteoporotic vertebral fracture (OVF) patients.

**Methods:**

Based on the presence or absence of FI, 223 OVF patients were divided into two groups. The demographics of patients with and without FI were compared. The visual analogue scale and Oswestry disability index scores were compared preoperatively and after PKP treatment between these groups.

**Results:**

Thoracolumbar fascia injuries were observed in 27.8% of patients. Most FI showed a multi-level distribution pattern which involved a mean of 3.3 levels. Location of fractures, severity of fractures and severity of trauma were significantly different between patients with and without FI. In further comparison, severity of trauma was significantly different between patients with severe and non-severe FI. In patients with FI, VAS and ODI scores of 3 days and 1 month after PKP treatment were significantly worse compared to those without FI. It showed the same trend in VAS and ODI scores in patients with severe FI when compared to those patients with non-severe FI.

**Conclusions:**

FI is not rare in OVF patients and presents multiple levels of involvement. The more serious trauma suffered, the more severe thoracolumbar fascia injury presented. The presence of FI which was related to residual acute back pain significantly affected the effectiveness of PKP in treating OVFs.

**Trial registration:**

retrospectively registered.

## Background

Osteoporosis has become a major public health problem worldwide as an aging society is coming [1]. Osteoporotic vertebral fracture (OVF) characterized by significant back pain and limited mobility is one of the most severe complications of osteoporosis [2–4]. Different from traumatic spinal fractures in young adults, OVF is always caused by low energy trauma, even though patients did not present any trauma history [5]. Magnetic resonance imaging (MRI) is most effective in detecting OVF which typically presents bone marrow edema within the vertebral body [6]. However, in our clinical practice, except for bone edema, posterior fascia edema was also usually presented in several patients. Thoracolumbar fascia edema with extensive or localized areas indicated posterior peri-vertebral soft tissue injury. Such fascia injury (FI), however, is rarely discussed in OVF patients in previous literatures. To date, the clinical characteristics of such patients and features of thoracolumbar fascia injury are still unclear.

Vertebral augmentation techniques, known as percutaneous kyphoplasty (PKP) or vertebroplasty (PVP), have been widely used to treat OVF in recent years. To date, controversy still exists considering vertebroplasty in addressing acute pain caused by OVF. High- to moderate- quality evidence showed that compared to sham procedure, vertebroplasty presented no important benefit in terms of pain, disability and quality of life in the treatment of acute or subacute OVF [7, 8]. In contrast, a number of studies reported that vertebroplasty and kyphoplasty should be the best choice in treating OVF. Significant pain relief and early mobilization could be achieved after PKP or PVP [9–12]. However, several patients still presented residual thoracolumbar back pain after vertebral augmentation surgery [13, 14]. Rib fracture, subsequent new vertebral fracture, osteoporotic ostalgia, and other factors were reported to be related to residual pain after PKP or PVP [15, 16]. Thoracolumbar fascia injury which indicated posterior vertebral soft tissue damage should be a potentially crucial factor for residual back pain [15, 17]. Nonetheless, it is rarely discussed whether thoracolumbar fascia injury is closely related to residual back pain and whether fascia injury will affect the therapeutic effect of PKP or PVP in treating OVF is not yet clear. Thus, we performed this study to describe the characteristics of fascia injury in OVF patients and further evaluate its clinical significance in OVF patients treated by vertebral augmentation techniques. 

## Methods

### Study population

This was a retrospective study approved by the Institutional Review Board of The First Affiliated Hospital of Soochow University, and informed consent was obtained from all patients.

Between January 2019 to December 2020, 223 patients (185 females and 38 males) who were diagnosed as single level OVF and treated by PKP surgery in our institution were included in this study. The included patients were classified into two groups according to the presence or absence of fascia injury (With-FI group and Without-FI group). The average age of the patients was 70.35 ± 8.34 years (ranging from 55 to 93 years).

Inclusion criteria: 1) bone marrow edema in the affected vertebra with or without posterior fascia injury in MR images; 2) severe back pain unresponsive to conservative treatment for at least one week; 3) T-scores assessed by Dual Energy X-ray Absorptiometry was less than -2.5.

Exclusion criteria: 1) pathological fracture secondary to infection or malignancy; 2) unable to tolerate the operation; 3) severe OVF presented fracture dislocation; 4) neurological deficit caused by OVF; 5) combined with Alzheimer's disease or other diseases which made the patients unable to finish information collection on their own.

The time interval between fracture and PKP surgery was recorded as fracture age. The OVF related trauma history was evaluated and the severity of trauma was divided into three degrees (none or minor, moderate, and severe). Vertebral fractures caused by coughing, twisting waist, and other daily activities were considered minor trauma. Moderate trauma was defined as stumble, falls from chairs, and other minor accidents caused by definite external forces. Serious trauma was referred to car accidents, falls from high places and other damages caused by severe external forces.

### Surgical procedure

PKP surgeries were performed under general anesthesia in the prone position according to the standard procedure reported in the previous study [18]. After fracture reduction by the inflatable balloon expansion and then polymethylmethacrylate (PMMA) cement was injected into the vertebral body under fluoroscopic guidance to stabilize the fractured vertebrae. Patients were allowed to ambulate one day after surgery. Anti-osteoporotic medications, including calcium, vitamin D3 and bisphosphonates, were routinely used in all the including patients during follow-up time.

### Clinical and radiological evaluation

Symptoms of pain were assessed by Visual analogue scale (VAS) scores and patients’ function was evaluated by Oswestry disability index (ODI) scores. VAS scores ranged from zero to ten grades which zero grade indicating no pain at all and ten grade indicating unbearable and drastic pain. Sexual function was eliminated when ODI score evaluation was performed. Results were recorded preoperatively, three days after surgery, one month, three months after surgery, and at the last follow-up.

**Fig. 1:**
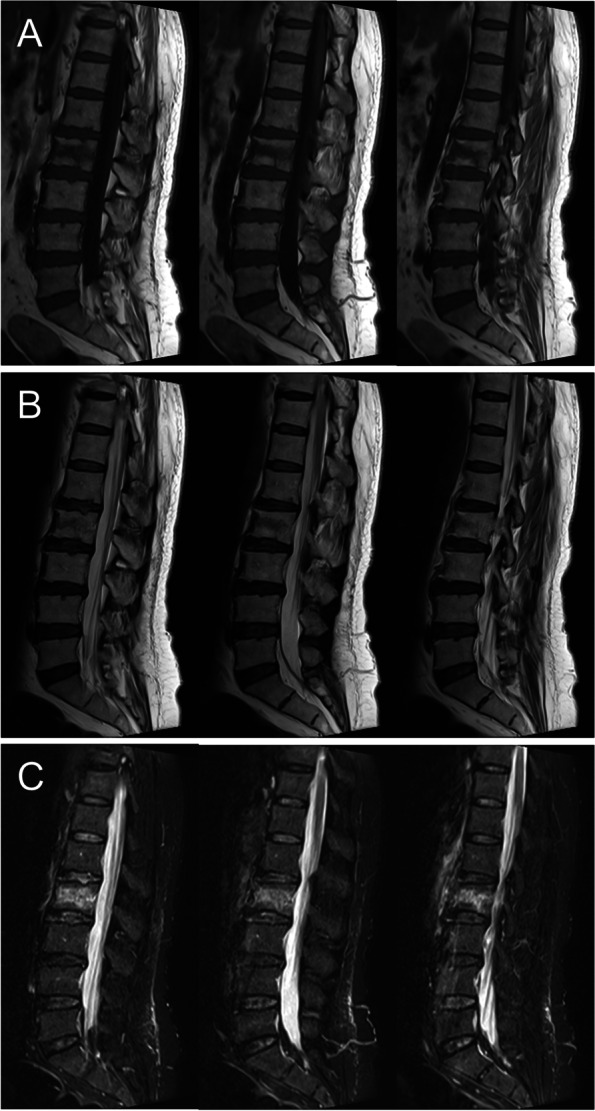
Osteoporotic vertebral fracture with non-severe fascia injury. Three most central slices of sagittal T1-weighted (**A**), T2-weighted (**B**) and T2-weighted fat suppression (**C**) MR images show L2 vertebral fracture with non-severe FI

**Fig. 2:**
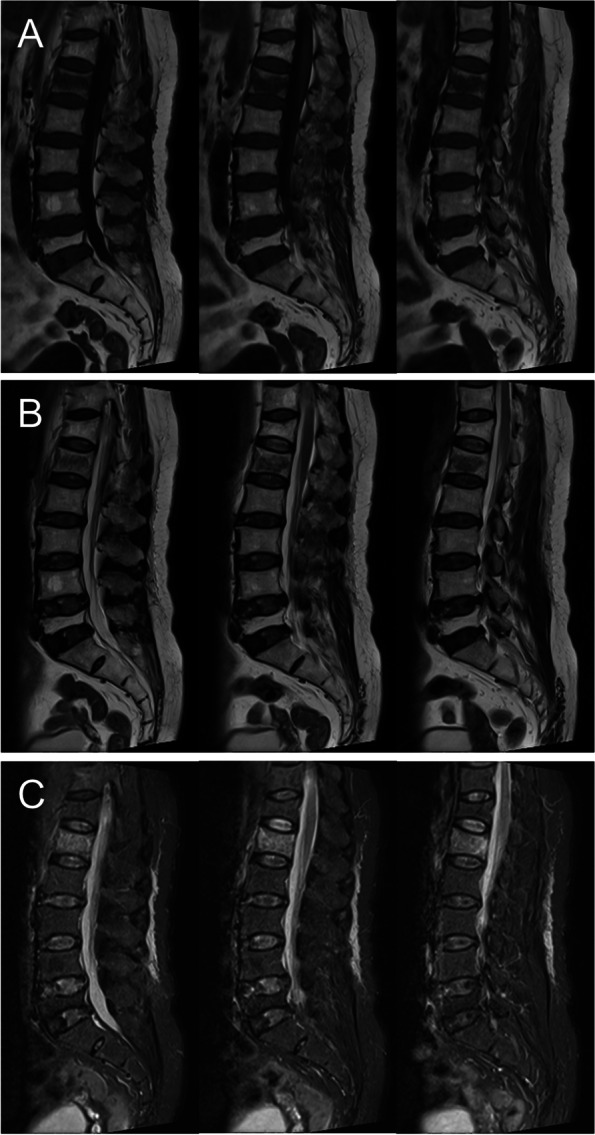
Osteoporotic vertebral fracture with severe fascia injury. Three most central slices of sagittal T1-weighted (**A**), T2-weighted (**B**) and T2-weighted fat suppression (**C**) MR images show L1 vertebral fracture with severe FI

The involved location of the fracture was assessed according to preoperative MR and X-ray images. The fracture location was recorded as thoracic (T5-T10), thoracolumbar (T11-L2), or lower lumbar region (L3-L5). Fracture severity which was divided into four degrees was assessed by the method of Genant et al. [19]. Thoracolumbar fascia injury was defined based on MR findings of fascia edema and focal tenderness on physical examination in the corresponding level of fascia edema. Two senior surgeons independently evaluated the MR images in PACS system (Neusoft Inc., Shenyang, Liaoning, China) and the final decision was made through consensus discussion in case of disagreement. The radiological features of FI were analyzed mainly on the three most central slices of the sagittal MR images. The involved locations of FI were recorded as thoracolumbar (T11-L2), lower lumbar (L3-L5), or both thoracolumbar and lower lumbar regions. The length of the fascia edema signal was recorded by calculating the number of involved levels of FI. Then patients with FI were further divided into Non-severe (≤ three levels) and Severe (**> **three levels) FI groups (Figs. 1 and 2).

### Statistical analysis

The differences between the groups (With-FI vs. Without-FI; Non-severe FI vs. Severe FI) were compared by the t test or Mann–Whitney U test for continuous variables and Chi-square test for categorical variables. Paired t test or Wilcoxon signed-rank test was used to compare the differences at different follow-up time. The probability value of less than 0.05 was considered statistically significant. SPSS 19.0 software (SPSS Inc, Chicago, IL, USA) was used to perform the statistical analysis.

## Results

**Fig. 3:**
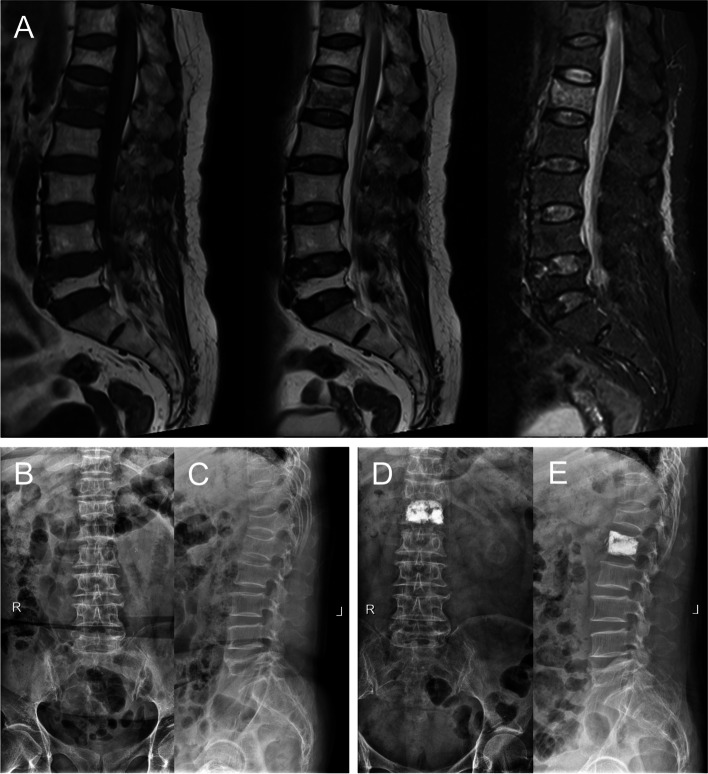
A 59-year-old female patient with back pain for 10 days. The T1-weighted, T2-weighted and T2-weighted fat suppression MR images (**A**) show L1 vertebral fracture with severe FI. Preoperative anteroposterior (**B**) and lateral (**C**) X-ray images show L1 and T12 vertebral compression. Postoperative anteroposterior (**D**) and lateral (**E**) X-ray images present satisfactory cement distribution after percutaneous kyphoplasty surgery

The demographic data of the included patients were listed in Table 1. In this study, 62 patients were detected with fascia injury (With-FI group) while 161 patients had no fascia injury (Without-FI group) according to the preoperative MR images which represented that the incidence of FI was 27.8% in our OVF patients. Then, in With-FI group, 23 patients were further classified into Severe FI group and 39 patients were considered as Non-severe FI group based on severity of FI. All PKP surgeries were performed successfully without severe complications (Fig. 3). Patients were followed up for 17.27 ± 5.19 months on average.

**Table 1:** Demographic data for With-FI group and Without-FI group

	**With FI (*n* = 62)**	**Without FI (*n* = 161)**	***P* value**
**Age** (year)	71.32 ± 8.73	69.98 ± 8.18	0.28
**Gender** (female)	53/62	132/161	0.53
**BMI** (kg/m^2^)	24.83 ± 3.43	25.35 ± 2.91	0.29
**BMD** (T score)	-3.09 ± 0.34	-3.01 ± 0.36	0.12
**Glucocorticoid usage**	4/62	8/161	0.91
**Smoking**	4/62	13/161	0.68
**Daily alcohol consumption**	3/62	8/161	1.00
**Fracture age** (day)	10.82 ± 4.23	9.77 ± 3.67	0.08
**Fracture location**			0.04*
Thoracic	2	15	
Thoracolumbar	46	128	
Lower lumbar	14	18	
**Severity of fracture**			0.01*
< 25%	30	92	
25%-50%	24	57	
50–75%	4	12	
> 75%	4	0	
**Severity of trauma**			< 0.001*
none or slight	14	107	
moderate	43	46	
severe	5	8	

The continuous variables are expressed as mean ± standard deviation; the categorical variables are expressed as frequency

*FI* Fascia injury

^*^Statistical significance was defined as *P* < 0.05

**Fig. 4:**
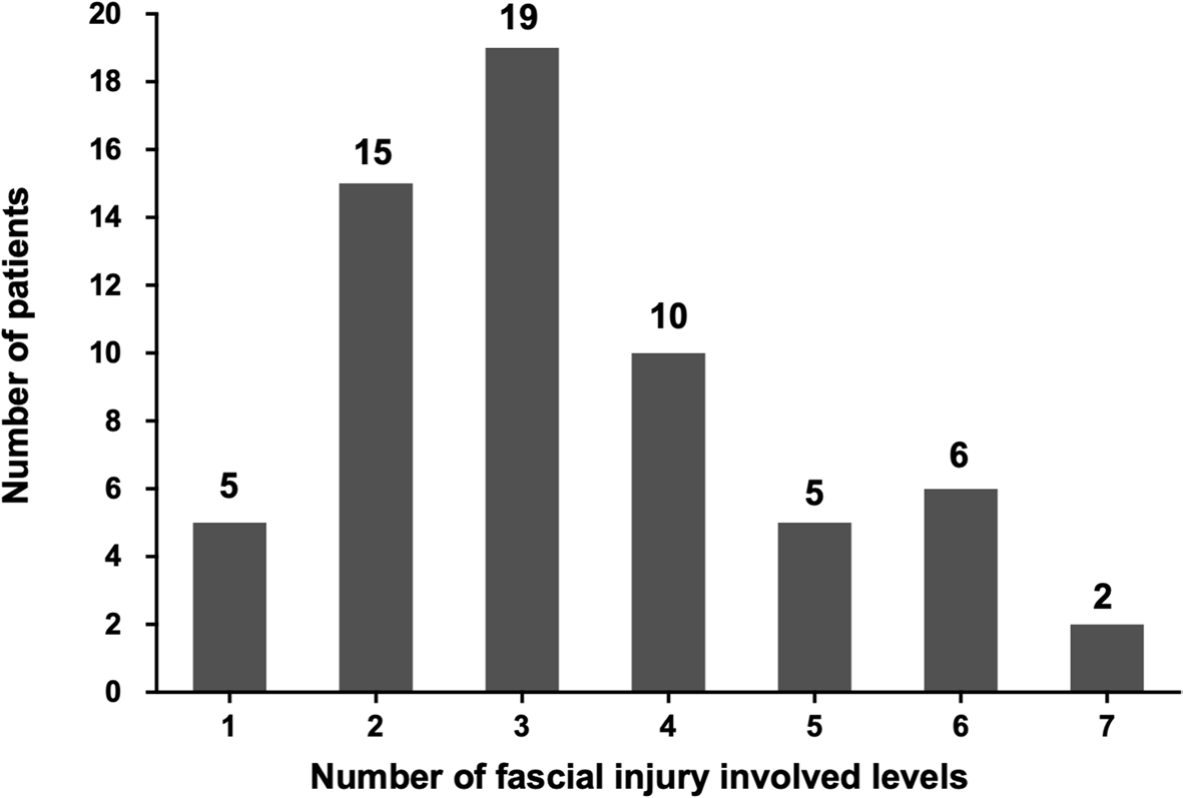
The involved levels of fascia injury in osteoporotic vertebral fracture patients

Most of the fascia injuries showed a multi-level distribution pattern which involved a mean of 3.3 levels (ranging from one to seven levels) (Fig. 4).

**Fig. 5:**
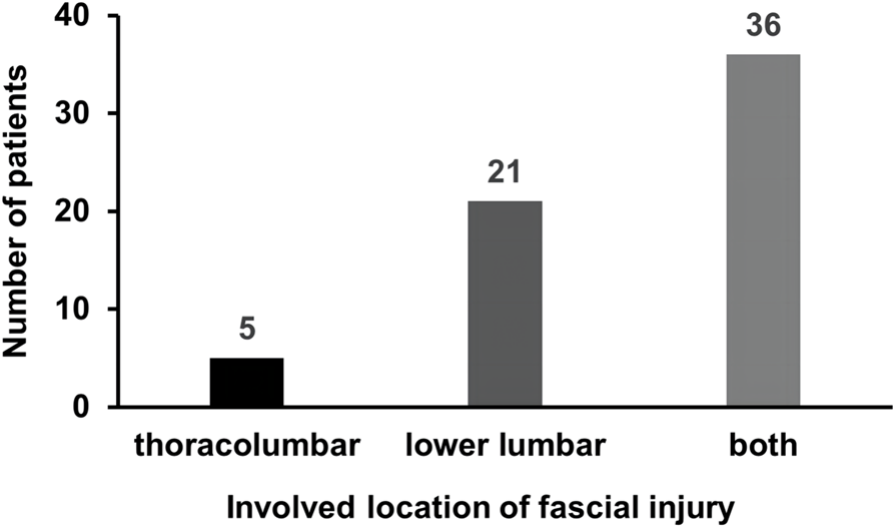
The involved locations of fascia injury in osteoporotic vertebral fracture patients

The involved locations of FI were: 21 cases occurred in the lower lumbar region (L3-L5), 5 cases in the thoracolumbar region (T11-L2), and 36 cases involved both thoracolumbar and lower lumbar regions (T11-L5); none was found in the thoracic region (T5-T10) (Fig. 5).

There were no statistically significant differences between patients with FI and those without FI in terms of patients’ age, gender, BMI, BMD, fracture age, and other factors (Table 1).

However, statistically significant differences were detected in fracture location (*P* = 0.04), severity of fracture (*P* = 0.01), and severity of trauma (*P* < 0.001) between the two groups. OVF patients with FI usually suffered more serious trauma than those without FI (48/62, 77.42% vs 54/161, 33.54%; *P* < 0.001); the severity of fracture was also more serious in With-FI group than that in Without-FI group (8/62, 12.9% vs. 12/161, 7.45%; *P* = 0.01). In addition, in With-FI group, more fractures were located in lower lumbar region than those in Without-FI group (14/62, 22.58% vs. 18/161, 11.18%; *P* = 0.04). A comparison between Severe and Non-severe FI groups showed that the severity of trauma in Severe FI group was much more serious than that in Non-severe FI group (P < 0.05) (Table 2).

**Table 2:** Demographic data for None-severe FI group and Severe FI group

	**Non-severe FI** **(≤ 3 levels, *n* = 39)**	**Severe FI** **(> 3 levels, *n* = 23)**	***P* value**
**Age** (year)	70.87 ± 8.54	72.09 ± 9.19	0.60
**Gender** (female)	35/39	18/23	0.39
**BMI** (kg/m^2^)	24.82 ± 3.50	24.84 ± 3.40	0.99
**BMD** (T score)	-3.06 ± 0.35	-3.16 ± 0.33	0.27
**Glucocorticoid usage**	3/39	1/23	1.00
**Smoking**	2/39	2/23	0.99
**Daily alcohol consumption**	1/39	2/21	0.64
**Fracture age** (day)	10.85 ± 4.34	9.26 ± 3.52	0.14
**Fracture location**			0.80
Thoracic	1	1	
Thoracolumbar	30	16	
Lower lumbar	8	6	
**Severity of fracture**			0.27
< 25%	16	14	
25%-50%	16	8	
50–75%	3	1	
> 75%	4	0	
**Severity of trauma**			0.03*
none or slight	12	2	
moderate	26	17	
severe	1	4	

The continuous variables are expressed as mean ± standard deviation; the categorical variables are expressed as frequency

*FI* Fascia injury

^*^Statistical significance was defined as *P* < 0.05

The preoperative VAS and ODI scores did not differ significantly between With-FI group and Without-FI group. After PKP treatment, both VAS and ODI scores were improved when compared with preoperative values in both groups (Table 3).

**Table 3:** VAS and ODI scores of OVF patients With and Without FI evaluated before and after PKP surgery

	**With FI (*n* = 62)**	**Without FI (*n* = 161)**	***P* value**
**VAS**			
preoperative	7.63 ± 0.61	7.70 ± 0.63	0.48
3 days after surgery	3.71 ± 0.76	2.97 ± 0.66	< 0.01*
1 month after surgery	3.48 ± 0.65	2.83 ± 0.58	< 0.01*
3 months after surgery	2.16 ± 0.55	2.14 ± 0.52	0.82
last follow-up	2.11 ± 0.55	2.11 ± 0.52	0.99
**ODI**			
preoperative	70.94 ± 2.80	71.00 ± 3.02	0.88
3 days after surgery	38.89 ± 7.19	28.61 ± 7.35	< 0.001*
1 month after surgery	36.79 ± 7.38	28.67 ± 6.95	< 0.001*
3 months after surgery	20.47 ± 4.34	21.24 ± 4.75	0.27
last follow-up	20.50 ± 4.09	20.68 ± 4.47	0.78

The continuous variables are expressed as mean ± standard deviation

*FI* Fascia injury, *VAS* Visual analog scale, *ODI* Oswestry disability index

^*^Statistical significance was defined as *P* < 0.05

However, the VAS and ODI scores three days and one month after surgery were both significantly worse in With-FI group than those values in Without-FI group (*P* < 0.01). Then there were no statistically significant differences in the VAS and ODI scores between the two groups three months after surgery and at the last follow-up. Further comparison between Severe and Non-severe FI groups showed the VAS and ODI scores were also much worse in Severe FI group than those in Non-severe group three days and one month after surgery (*P* < 0.05). Then the VAS and ODI scores showed no statistically significant differences three months after surgery and at the last follow-up (Table 4).

**Table 4:** VAS and ODI scores of OVF patients with Severe and Non-severe FI evaluated before and after PKP surgery

	**Non-severe FI** **(≤ 3 levels, *n* = 39)**	**Severe FI** **(> 3 levels, *n* = 23)**	***P* value**
**VAS**			
preoperative	7.62 ± 0.59	7.65 ± 0.65	0.82
3 days after surgery	3.49 ± 0.60	4.09 ± 0.85	0.002*
1 month after surgery	3.28 ± 0.51	3.83 ± 0.72	0.001*
3 months after surgery	2.15 ± 0.54	2.17 ± 0.58	0.89
last follow-up	2.13 ± 0.47	2.09 ± 0.67	0.78
**ODI**			
preoperative	70.46 ± 2.65	71.74 ± 2.91	0.08
3 days after surgery	36.87 ± 4.51	42.30 ± 9.41	0.015*
1 month after surgery	34.51 ± 5.82	40.65 ± 8.24	0.001*
3 months after surgery	20.00 ± 4.29	21.26 ± 4.39	0.27
last follow-up	20.08 ± 4.31	21.22 ± 3.68	0.29

The continuous variables are expressed as mean ± standard deviation

*FI* Fascia injury, *VAS* Visual analog scale, *ODI* Oswestry disability index

^*^Statistical significance was defined as *P* < 0.05

## Discussion

Osteoporotic vertebral fracture combined with thoracolumbar fascia injury is not a rare phenomenon. In this retrospective study, we found its incidence to be 27.8% (62/223). A recent study by Wang et al. [15] described fascia injury in 7.4% (16/215) of the patients with OVF. In another prospective cohort study by Yan et al*. *[11], they evaluated the cause of residual back pain after vertebroplasty and they reported that the prevalence of fascia injury was as high as 42.1%. Thus, FI should be considered common complicated damage in OVF patients which is usually neglected in clinical work.

Thoracolumbar fascia is a complex arrangement of layers of fascia and aponeurosis, located in the thoracic and lumbar segments of the spine. Thoracolumbar fascia plays an important biomechanical role in maintaining the stability of the lumbar spine and pelvis and the damaged fascia usually caused local back pain [20]. In this study, FI mainly occurred in thoracolumbar vertebral fracture patients and FI tends to occur in lower lumbar region or extend from thoracolumbar to lower back region which may be attributed to the protective mechanism of the fascia under spinal trauma and downward transmission of spinal mechanical load [21, 22]. The lower lumbar fascia injury may partially explain the interesting phenomenon that several osteoporotic thoracolumbar vertebral facture (T11-L2) patients usually complain of distal lumbosacral pain which is still vague in previous literatures [23].

OVFs are mostly caused by minor trauma or even no trauma due to the significant loss of vertebral bone quality and quantity [24, 25]. However, as presented in this study, when OVF is combined with FI, these patients would generally suffer from moderate or more serious trauma. Furthermore, the severity of fracture was also related to the occurrence of FI in this study. We further found that most FI showed a multi-level distribution pattern (a mean of 3.3 levels) and the severity of FI (> 3 levels) was positively correlated with the degree of trauma. These could be explained that the more serious trauma the patients suffered, the more severe fracture and fascia injury would develop. Thoracolumbar fascia injury should be considered an indicator of serious trauma and severe vertebral fracture in OVF patients.Furthermore, it is interesting that for those OVFs with FI, more fractures were located in lower lumbar region than those without FI (22.58% vs. 11.18%) in this study. This may be explained to the fact that osteoporotic lower lumbar fractures are usually caused by more severe trauma which has a higher risk of developing fascia injury [26].

Though there was controversy in the past, PKP is becoming the standard procedure for the treatment of OVFs nowadays [8, 9, 11, 27, 28]. By stabilizing the fractured vertebra using bone cement, significant pain relief and early mobilization could be achieved after surgery. However, a portion of patients still complained of residual back pain after PKP or PVP surgery and were considered unsatisfied about being performed surgery [15, 29]. According to the literatures, residual back pain was rarely discussed and unsatisfactory cement distribution, severe paraspinal muscle degeneration or fascia injury, and depression were reported to be the risk factors for residual back pain [15, 16, 30]. A previous study reported that OVFs with concomitant thoracolumbar FI did not respond well to vertebral augmentation surgeries, indicating that FI should be related to residual back pain [17]. In our study, the postoperative VAS and ODI scores were both significantly worse in OVF patients with FI than those without FI (*P* < 0.05), which indicated that the presence of FI affected the effectiveness of PKP in treating OVFs. Further comparison between Severe and Non-severe FI groups presented that the more severe injury of thoracolumbar fascia (> 3 levels), the more significant residual pain would have. The plausible explanation for these results is that the thoracolumbar fascia is abundant in nerve endings [15]; though vertebral augmentation surgeries stabilized fractured vertebra and relieved fracture related pain, however, the damaged fascia and soft tissue edema was the other painful source of low back pain.

There were several limitations in this study. First, the severity of trauma was not defined quantitatively and subjective bias might exist based on the recount from patients. Second, we defined fascia injury as the high intensity lesion around fascia in MR images which may not be accurately indicate fascial fiber injury. Third, the mechanism of occurrence of fascia injury in OVF patients could not be elucidated in this study. Further biomechanical studies and finite element analysis should be performed to explain it. Furthermore, the degeneration of paraspinal muscle which was not evaluated in this study might be another factor that related to the severity of fascia injury in OVF patients [31, 32]. Further prospective controlled studies are needed to further explore the clinical significance of fascia injury in OVF patients treated by PKP or PVP due to the retrospective nature of this study.

## Conclusions

Thoracolumbar fascia injury is not rare and should be regarded as important concomitant damage in OVF patients. FI is mainly located in the lower lumbar region with multiple levels involved. The more serious trauma suffered, the more severe thoracolumbar fascia injury presented. The presence of FI which was related to residual back pain significantly affected the effectiveness of PKP in treating OVFs.

## Data Availability

Data and materials included in the study are available from the corresponding author on request.

## Abbreviations

OVF: Osteoporotic vertebral fracture
FI: Fascia injury
MRI: Magnetic resonance imaging
PKP: Percutaneous kyphoplasty
PVP: Percutaneous vertebroplasty
VAS: Visual analogue scale
ODI: Oswestry disability index
